# Attachment Styles and Personal Growth following Romantic Breakups: The Mediating Roles of Distress, Rumination, and Tendency to Rebound

**DOI:** 10.1371/journal.pone.0075161

**Published:** 2013-09-16

**Authors:** Tara C. Marshall, Kathrine Bejanyan, Nelli Ferenczi

**Affiliations:** Department of Psychology, Brunel University, London, United Kingdom; Catholic University of Sacred Heart of Rome, Italy

## Abstract

The purpose of this research was to examine the associations of attachment anxiety and avoidance with personal growth following relationship dissolution, and to test breakup distress, rumination, and tendency to rebound with new partners as mediators of these associations. Study 1 (*N* = 411) and Study 2 (*N* = 465) measured attachment style, breakup distress, and personal growth; Study 2 additionally measured ruminative reflection, brooding, and proclivity to rebound with new partners. Structural equation modelling revealed in both studies that anxiety was indirectly associated with greater personal growth through heightened breakup distress, whereas avoidance was indirectly associated with lower personal growth through inhibited breakup distress. Study 2 further showed that the positive association of breakup distress with personal growth was accounted for by enhanced reflection and brooding, and that anxious individuals’ greater personal growth was also explained by their proclivity to rebound. These findings suggest that anxious individuals’ hyperactivated breakup distress may act as a catalyst for personal growth by promoting the cognitive processing of breakup-related thoughts and emotions, whereas avoidant individuals’ deactivated distress may inhibit personal growth by suppressing this cognitive work.

## Introduction

There are wide individual differences in reactions to romantic breakups, with some people reporting little psychological or somatic disturbance, and others experiencing intensified sadness and anger [1], negative cognitions [2], a decline in life satisfaction [3], poorer physical health [4], and the onset of mental disorders such as major depression [5]. Although individual differences in breakup distress are well-established, few studies have examined the personality traits that predict personal growth following the end of a relationship – the potential silver lining of breaking up [6]. Notwithstanding the ubiquity of attachment theory for understanding the formation, maintenance, and termination of romantic relationships, no previous research has linked individual differences in attachment style to post-breakup growth. The present studies sought to fill this research gap and further explore the mediating role of breakup distress, rumination, and tendency to rebound with new partners. We begin by discussing attachment theory as a framework for understanding individual differences in post-breakup distress and personal growth.

### Attachment Theory

According to attachment theory, an infant’s history of interactions with caregivers shapes internal working models of self and other that guide affect, cognition, and behaviour throughout one’s life [7]–[9]. When caregivers are consistently available and responsive, individuals are likely to develop a secure attachment style, characterized by confidence that one is worthy of love, reliance on an attachment figure as a secure base from which to explore the world, and seeking proximity, comfort, and support from caregivers when feeling distressed [10]. This outsourcing of negative affect allows secure individuals to effectively regulate their emotions [11].

Insecurely attached individuals, on the other hand, tended to have caregivers who were inconsistently available and responsive (attachment-anxious individuals) or neither available nor responsive (attachment-avoidant individuals). As adults, highly anxious individuals tend to doubt their own worth and lovability, fear rejection, and seek excessive reassurance, approval, and closeness [12]. When caregivers are perceived as unavailable, anxious individuals tend to use hyperactivating strategies – such as crying, pleading, clinging, or throwing a tantrum – to restore proximity [13]. Conversely, highly avoidant adults are excessively self-reliant, mistrustful of others, and uncomfortable with intimacy [14]. They tend to use deactivating strategies when attachment figures are perceived as unavailable, which aim to restore self-sufficiency by defensively inhibiting distress and proximity-seeking [15]. Attachment avoidance and anxiety are commonly conceptualized as two orthogonal dimensions, with the low ends of each representing attachment security [16]. Germane to the present research, individual differences in attachment style predict reactions to relationship loss.

### Attachment Styles and Emotional Adjustment after Relationship Loss

Bowlby [17] proposed that reactions to relationship loss typically progress through three stages: protest, which includes crying, anger, disbelief, and attempts to re-establish contact and proximity with the lost attachment figure; despair and sadness; and, eventually, the reorganization of one’s attachment hierarchy and detachment. Reorganization occurs by upgrading new or existing partners, downgrading ex-partners, or maintaining a functional symbolic attachment if the partner is deceased. Along related lines, Stroebe and Schut [18] proposed that coping with relationship loss entails oscillation between two processes: working through the loss to extract meaning, and down-regulating emotional disruption to restore everyday functioning. These two processes require attachment system hyperactivation and deactivation, respectively [13].

Secure, anxious, and avoidant attachment styles are differentially related to post-breakup emotional adjustment. Secure individuals tend to face relationship breakups with greater resilience, acceptance, and emotional recovery than do insecure individuals [19], [20]. Highly anxious individuals, compared to less anxious individuals, tend to respond to breakups with hyperactivated emotional and physiological distress, preoccupation with ex-partners, drug and alcohol abuse, and a lost sense of identity [1], [21]–[25]. These findings are consistent with Bowlby’s [17] observation that anxious individuals are more susceptible to *chronic mourning* – prolonged protest, despair, and continued attachment to the lost partner. Anxious individuals’ amplified breakup distress has been attributed to their poor coping strategies, maladaptive rumination, dysfunctional reliance on an ex-partner to provide a safe haven, and their tendency to blame themselves for negative events [21], [23], [24], [26], [27].

In contrast, highly avoidant individuals tend to be lower in non-marital breakup distress than less avoidant individuals [28], consistent with their tendency to deactivate attachment-related thoughts and emotions [15]. Indeed, Bowlby [17] held that avoidant individuals show an absence of grief in response to relationship loss – little protest and despair, and quick progression to the reorganization/detachment phase. Davis and colleagues [21], however, found that avoidance was related to a number of negative reactions to a breakup, such as greater self-blame and use of drugs and alcohol to cope, lower motivation to replace the ex-partner with a new partner, and less interest in sex. Furthermore, Birnbaum and colleagues [26] found that avoidant individuals showed poorer post-divorce emotional adjustment and well-being compared to secure individuals, suggesting that significant relationship threats, such as divorce, may penetrate the habitual emotional defences of avoidant individuals. We examined whether the intensity of anxious and avoidant individuals’ breakup distress predicted their personal growth.

### Personal Growth Following Relationship Dissolution

Personal growth, variously termed stress-related or posttraumatic growth, refers to the positive life changes people make in reaction to negative life events [29]. These changes tend to focus on cultivating one’s character strengths, sense of meaning in life, and connectedness with others [30]. Accordingly, Tashiro and Frazier [6] found that after a romantic breakup, people reported positive growth in their personal traits (e.g., greater self-confidence and independence), relationship-maintenance behaviours (e.g., better communication skills), environment (e.g., cultivating better relationships with friends and family, focusing more on school or work), and expectations of future romantic partners. These authors also found that post-breakup growth was greater in women, highly agreeable individuals, and those who attributed the cause of the breakup to external factors rather than to the self. Other studies have found that women who are separated or divorced report experiencing greater personal growth than women who are married [31], consistent with research suggesting that divorce may motivate personal growth [32].

Importantly, none of these studies explored the association of attachment style with personal growth, nor focused on breakup distress as a catalyst for the cognitive processing that promotes personal growth. Related findings from the posttraumatic growth literature have linked attachment security with greater posttraumatic growth than attachment insecurity [33], but this research focused on people who experienced extreme trauma (former political prisoners exposed to torture), whereas we have focused on people coping with romantic breakups. Importantly, other findings suggest that the distress experienced at the time of trauma is positively associated with later growth [34], [35], suggesting that attachment-anxious individuals’ propensity towards intensified distress, and attachment-avoidant individuals’ tendency to suppress their distress, may have implications for their post-breakup growth.

### The Present Research

In Studies 1 and 2, we predicted that attachment anxiety would be positively associated with breakup distress and, in turn, with greater personal growth. In contrast, we predicted that attachment avoidance would be negatively associated with breakup distress and, in turn, with less personal growth. Study 2 further investigated whether the association of breakup distress with personal growth might be accounted for by ruminative reflection, brooding, and the tendency to rebound with new romantic partners. Both studies also compared participants whose breakups occurred longer ago (approximately one year) with those whose breakup was more recent (less than one year), reasoning that breakup distress and rumination may be stronger predictors of personal growth when breakups occurred longer ago; the passage of time might allow the acute negative emotions experienced immediately after a breakup to subside enough to work through the loss and extract meaning. Insofar as highly anxious people experience greater breakup distress than avoidant individuals, they may require more time to digest the breakup and develop a growth-promoting narrative. Examining the influence of length of time since the breakup occurred therefore allowed us to gauge the time course by which breakup distress may catalyze personal growth. Overall, in contrast to the focus on emotional adjustment and recovery in the breakups literature, we have focused on individual differences in a relatively unstudied outcome, personal growth.

## Study 1

### Ethics Statement

Ethical approval for Studies 1 and 2 was obtained from the Brunel University Psychology Research Ethics Committee. Participants provided written informed consent at the beginning of the survey, and all responses were confidential. Anonymized data for Studies 1 and 2 is available by request from the first author.

### Participants

The sample for Study 1 consisted of 411 participants (273 women, 136 men, 2 unspecified; *M*_age_ = 23.47, *SD* = 6.75). They were recruited through posting a link to an online survey on three websites that host online psychology surveys (Social Psychology Network Online Social Psychology Studies, Psychology on the Net, and the intranet at the authors’ university) and through the personal contacts of the research assistant. A significantly higher proportion of women than men (54% versus 43%) were currently involved in a relationship, x^2^(1, *N* = 409) = 5.83, *p* = .02. Of the currently-involved participants, 62% were exclusively dating their current partner, 13% were cohabitating, 13% were married, 8% were non-exclusively dating their current partner, and 4% were engaged. Current involvement in a relationship was effect-coded to assess its associations with the other variables (1 = currently involved, −1 = not currently involved). The average length of the current relationship was 140.03 weeks (*SD* = 175.39). 47% of participants were American, 26% were British, 7% were European, 5% were Latin American, 3% were South Asian, 3% were East or Southeast Asian, 2% were African, 2% were Caribbean, 2% were Middle Eastern, 2% were Canadian, and 2% were from Australia or New Zealand.

### Procedure and Materials

Participants completed the measures in the order listed below to encourage chronological recollection of the breakup, subsequent distress, and personal growth. Several questions at the end of the survey addressed demographic variables, current relationship status and, if involved, current relationship length. In both studies, we also measured self-esteem with the 10-item Rosenberg Self-Esteem Inventory [36]. It was included as a control variable in the following regression analyses, but because it did not influence the pattern of results, it will not be mentioned further. Cronbach’s alpha coefficient for continuous scales is reported in Table 1.

**Table 1:** Study 1: Pearson’s correlations, descriptive statistics, and reliability coefficients.

Variable	1	2	3	4	5	6	7	8	9
1. Weeks since breakup	1.00								
2. Partner initiated	.11*	1.00							
3. Relationship length	.10*	−.04	1.00						
4. Currently involved	**.36**	.01	.11*	1.00					
5. Current distress	**−.26**	.12*	.01	**−.28**	1.00				
6. Anxiety	**−.27**	.15**	−.08	**−.29**	**.34**	1.00			
7. Avoidance	−.09^†^	−.05	−.12*	**−.31**	**.20**	**.42**	1.00		
8. Breakup distress	−.04	**.41**	.05	−.04	**.33**	**.35**	−.01	1.00	
9. Personal growth	−.03	−.05	.08^†^	.14**	−.17**	−.02	−.15**	.11*	1.00
Mean	141.29	–	100.07	–	0.00	50.48	43.03	54.34	70.31
*SD*	205.46	–	115.35	–	2.55	15.44	12.62	14.68	18.70
α	–	–	–	–	.81	.94	.92	.92	.94

*Note.* ^†^ *p*<.10. *p<.05. **p<.01. Bolded figures were significant at *p*<.0001. Weeks since breakup = how much time (in weeks) has elapsed since the breakup. Partner initiated = partner initiated the breakup. Relationship length = length (in weeks) of former relationship. Currently involved = currently involved in a relationship. Current distress = current distress felt about the breakup. Breakup distress = distress felt immediately after the breakup occurred.

### Attachment Style

The Experiences in Close Relationships – Revised (ECR-R) Questionnaire [16] measures the anxious and avoidant dimensions of attachment with18 items each. Responses were assessed with a 5-point Likert scale ranging from *Strongly Disagree* (1) to *Strongly Agree* (5). Examples of anxious and avoidant items are, “I am afraid that I will lose my partner’s love” and “I prefer not to be too close to romantic partners,” respectively. Principle components analyses of the ECR-R in Studies 1 and 2 revealed that the items cleanly loaded on their intended factor.

#### Information about the past relationship and breakup

Participants were asked to think back to the most distressing romantic breakup they had ever experienced, and complete the rest of the questionnaire in reference to this breakup. Participants indicated the status of the relationship before the breakup (non-exclusive dating, exclusive dating, cohabitating, engaged, or married), how long the relationship lasted, who initiated the breakup (“I did,” “My partner did,” or “We both did”), and how much time had elapsed since the relationship ended. To prime memories of feelings experienced at the time of the breakup, participants also wrote a description of the circumstances surrounding the end of the relationship.

#### Breakup distress

The 16-item Breakup Distress Scale (BDS) [37] was modified to ask participants to rate how they felt *immediately after* the breakup occurred, similar to the retrospective assessments of breakup distress in other studies [25], [28]. Example items are “I felt stunned or dazed over what happened” and “I felt like crying when I thought about the person.” The items were measured on a 5-point Likert scale ranging from *Not at all* (1) to *Very much* (5).

#### Current distress

Three questions addressed *current* feelings about the breakup: “How much distress do you currently feel concerning the breakup?” (1 = *None*, 5 = *A great deal*), “To what extent do you still have feelings for your ex-partner?” (1 = *No feelings at all*, 5 = *Strong feelings*) and “To what extent do you feel that you are over the breakup?” (1 = *Not at all over it*, 5 = *Completely over it*). The last question was reverse-scored. These items were standardized and summed to form a composite measure of current distress.

Preliminary analyses revealed that breakup distress and current distress overlapped to a greater extent for participants whose breakup occurred more recently (i.e., less than the median of 64.5 weeks since the breakup; *r* = .42, *p*<.0001) than for participants whose breakup occurred longer ago (i.e., more than the median; *r* = .26, *p* = .001), suggesting redundancy in these constructs when breakups were more recent. As such, we decided to focus on breakup distress rather than current distress in the analyses.

#### Personal growth

Similar to the approach taken by Tashiro and Frazier [6], the instructions of the Posttraumatic Growth Inventory (PTGI) [29] were modified to ask participants how much life change they had experienced as a result of the breakup. 21 items (e.g., “I developed new interests,” “I’m more likely to change things that need changing,” and “I discovered that I am stronger than I thought I was”) were assessed with a 5-point Likert scale anchored with *Not at all* (1) and *A great deal* (5).

### Results

#### Descriptive statistics

Means, standard deviations, zero-order correlations, and Cronbach’s alpha coefficients are reported in Table 1. Several gender differences were significant: women reported greater duration of their past relationship [*M*_women = _113.85, *SD* = 121.77; *M*_men_ = 73.68, *SD* = 84.82; *t*(396) = 3.38, *p* = .001], greater breakup distress [*M*_women = _55.95, *SD* = 14.74; *M*_men_ = 51.17, *SD* = 13.96; *t*(395) = 3.10, *p* = .002], and greater personal growth [*M*_women = _72.88, *SD* = 17.45; *M*_men_ = 65.50, *SD* = 19.88; *t*(407) = 3.84, *p*<.0001]. In terms of additional information about the former relationship, 77% of participants indicated that they had been exclusively dating their former partner, 7% had been non-exclusively dating, 7% had been cohabitating, 5% had been engaged, and 3% had been married. Furthermore, 58% of women and 48% of men indicated that their former partner initiated the breakup; this gender difference approached significance, x^2^(1, *N* = 408) = 3.19, *p* = .07. Initiator status was effect-coded to assess its correlations with the other variables (1 = partner initiated the breakup, −1 = I initiated/we both initiated the breakup).

As seen in Table 1, only some of the correlations were consistent with Baron and Kenny’s [38] preconditions for mediation. Attachment anxiety (independent variable) was significantly correlated with the mediator (breakup distress) but not with the dependent variable (personal growth). Attachment avoidance (the other independent variable) was significantly correlated with personal growth, but not with breakup distress. Shrout and Bolger (39) suggested that one of Baron and Kenny’s [38] preconditions – that the independent variable significantly predicts the dependent variable – should not be required when testing mediation. Nonetheless, we found that all of the preconditions were met when control variables were added to the regression models. Avoidance significantly predicted breakup distress (β = −.19, *p*<.0001) and personal growth (β = −.16, *p*<.01) when anxiety was included as a predictor in the regression model. These associations remained significant after several control variables (sex, age, self-esteem, initiator status, length of time since the breakup, current involvement, current distress) were included as covariates. Anxiety significantly predicted breakup distress (β = .44, *p*<.0001) but not personal growth when avoidance was included as a predictor; after the control variables were included as covariates, anxiety remained a significant predictor of breakup distress and was also a significant predictor of personal growth (β = .31, *p*<.0001). The latter finding suggests that one or several of the control variables helped to pull out the main effect of anxiety on personal growth.

We conducted structural equation modelling with AMOS 18 to assess the indirect effects of anxious and avoidant attachment on personal growth via breakup distress. We also tested whether breakup distress moderated rather than mediated the associations of attachment anxiety and avoidance with personal growth, but did not find any significant interaction effects. Moreover, none of the interactions of anxiety and avoidance with gender or with each other were significant, so they will not be mentioned further.

#### Structural equation models

Model fit was evaluated with the following indices: the chi-square statistic, which should be non-significant (though unrealistic to obtain with larger samples); the comparative fit index (CFI), which should be.95 or greater; the root-mean-square error approximation (RMSEA), which should be.06 or less; and the standardized root-mean-square residual (SRMR), which should be.08 or less. Nested models were compared with the chi-square difference test. Because AMOS 18 requires complete data, the following analyses were based on 364 participants (236 women, 126 men, 2 unspecified). In Studies 1 and 2, participants who were retained did not differ from those who were excluded due to missing data.

Item parcels were used to create latent variables. Parcels were created according to the recommendations of Russell, Kahn, Spoth, and Altmaeir [40]: exploratory factor analyses were first conducted for each scale and the items rank-ordered according to the size of their factor loadings. Items were then assigned to parcels in pairs, with the highest loading item paired with the lowest loading item, so that parcels equally reflected the latent variable. We created three parcels each for the latent variables of anxiety, avoidance, breakup distress, and personal growth. Consistent with recommendations for analyzing structural equation models [41], we tested the measurement model first, followed by competing structural models.

#### Measurement model

A confirmatory factor analysis indicated that the data provided a good fit to the measurement model [χ^2^(48) = 87.21, *p*<.0001, CFI = .99, RMSEA = .05 (CI = .03,.07), SRMR = .03]. The observed indicators all loaded significantly on the appropriate latent variable (*p*<.0001), indicating that they adequately measured the latent construct.

#### Structural model

**Figure 1:**
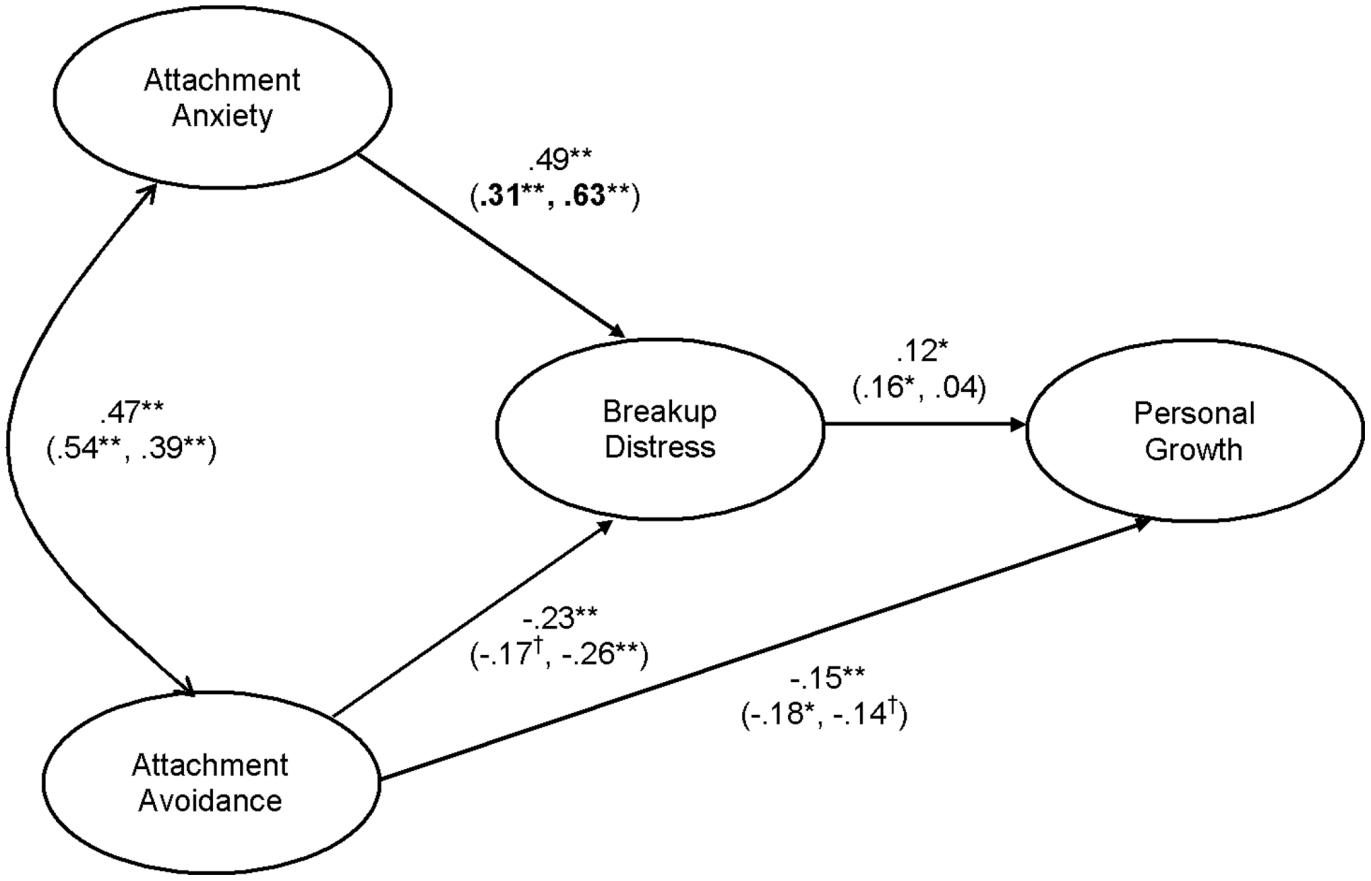
Study 1: Final model. The values within parentheses are the path coefficients for people whose breakup occurred longer ago (left side) and more recently (right side). Bolded values represent a significant group difference in the path coefficients.^ †^p<.10,*p<.05, **p<.01.

The initial test of the fully saturated structural model (i.e., all paths included) yielded the same fit indices as the test of the measurement model. All structural path coefficients were significant except for the path between anxiety and personal growth. A modified structural model that constrained this path to zero provided a good fit to the data [χ^2^(49) = 87.27, *p*<.0001, CFI = .99, RMSEA = .05 (CI = .03,.06), SRMR = .03], and did not significantly differ from the initial model [χ^2^D(1) = .06, *p*>.05]. Thus, the more parsimonious modified model that did not include this non-significant direct effect was retained. To test the direct effect of attachment avoidance on personal growth, the path from avoidance to personal growth was constrained to zero. The fit of this model was significantly worse than the model that included this path [χ^2^(50) = 94.83, *p*<.0001, CFI = .99, RMSEA = .05 (CI = .03,.07), SRMR = .05, χ^2^D(1) = 7.56, *p*<.05], suggesting that the direct effect of avoidance on personal growth was significant and should be retained. The standardized path coefficients of this final model are reported in Figure 1.

### Tests of Indirect Effects

Indirect effects in the final model were tested with a bootstrap procedure [39]. Examination of the 95% bias-corrected confidence intervals (CI) from 1,000 bootstrap samples revealed that the indirect effects of anxiety [*β* = .06, *p* = .05 (CI:.001,.12)] and avoidance [*β* = −.03, *p* = .03 (CI: −.07, −.002)] on personal growth through breakup distress were significant.

### Multiple-Group Comparison Analysis: Time since the Breakup

To test whether the final model fit the data similarly for participants whose breakups occurred longer ago (i.e., more than the median of 64.5 weeks; *N* = 171) versus more recently (less than the median; *N* = 174), we conducted a multiple-group comparison analysis with AMOS 18. Preliminary *t*-tests showed that the two groups did not significantly differ in breakup distress or personal growth. However, people whose breakup occurred more recently relative to those whose breakup occurred longer ago were significantly greater in anxiety [*M*s = 53.95 and 47.17, *SD*s = 14.66 and 15.41, respectively; *t*(373) = 4.36, *p*<.0001], avoidance [*M*s = 45.21 and 41.37, *SD*s = 11.97 and 12.84, respectively; *t*(368) = 2.98, *p*<.01] and current distress [*M*s = 7.75 and 5.59, *SD*s = 3.11 and 2.55, respectively; *t*(388) = 7.50, *p*<.0001].

First, multiple-group comparison analysis established that the factor loadings did not significantly differ across groups [χ^2^D(8) = 3.10, *p*>.05], verifying the invariance of the measurement model. Second, given equivalent factor loadings, the model in which the structural path coefficients were constrained to invariance across groups significantly differed from the model in which these paths were unconstrained [χ^2^D(4) = 11.84, *p = *.02], suggesting that at least one of the structural path coefficients was not equal across groups. Further examination revealed that only the path from anxiety to breakup distress was not equal [χ^2^D(1) = 9.64, *p*<.01]; the path coefficient was stronger for people whose breakups occurred more recently (*β* = .63, *p*<.01) than for people whose breakups occurred longer ago (*β* = .31, *p*<.01). Path coefficients for each group are reported in parentheses in Figure 1.

### Summary

The results of Study 1 indicated that anxious individuals’ heightened breakup distress fully accounted for their greater personal growth, whereas avoidant individuals’ lower breakup distress partially explained their lower personal growth. The multiple-group comparison analysis further revealed that the structural model depicted in Figure 1 fit the data more or less equally for people whose breakups occurred longer ago versus more recently. The only exception was that anxiety more strongly predicted breakup distress for people whose breakup occurred more recently than longer ago (we elaborate on this finding in the General Discussion). Overall, these results suggested that anxious individuals were more likely, and avoidant individuals less, to transform their breakup distress into personal growth, but the process by which this occurred was unclear. To clarify this process, we examined additional mediators in Study 2.

## Study 2

The goal of Study 2 was to replicate and extend the final model of Study 1 by testing three additional mediators of the association of breakup distress with personal growth: ruminative reflection, brooding, and the tendency to “rebound” with new partners after a breakup. Below we discuss each of these additional mediators and outline predictions for their associations with attachment style, breakup distress, and personal growth.

### Ruminative Reflection and Brooding

Rumination refers to a person’s repetitive thoughts about a past event [42], and includes adaptive and maladaptive subtypes (reflection versus brooding, preoccupation, and regrets, respectively) [23]. Similar to the “grief work” approach to loss, reflection involves introspection, cognitive reappraisal, and the construction of a meaningful narrative – a process that tends to facilitate emotional recovery [43]–[46]. More than promoting recovery from loss, however, reflection can also enhance personal growth [47].

Only a few studies have examined the link between attachment style, ruminative reflection, and emotional adjustment following a breakup. Notably, Saffrey and Ehrenberg [23] found that attachment anxiety was associated with less general reflection and, in turn, with poorer post-breakup emotional adjustment. These authors did not measure breakup-specific reflection, however. Other research has found that highly anxious individuals who engaged in greater breakup-specific reflection showed significantly *less* improvement in their emotional recovery compared to less anxious individuals, presumably because reflection may intensify the negative emotions that highly anxious people are so poor at regulating [27]. Still, the participants in this study were tested only one month after the breakup occurred; reflection may encourage highly anxious individuals’ recovery and growth only after more time has passed, allowing the initial breakup distress to subside. As such, we predicted that breakup-specific reflection would be positively associated with highly anxious individuals’ personal growth, but only when breakups occurred longer ago.

Research has also found that anxious attachment is associated with intrusive, maladaptive types of rumination – brooding and preoccupation – and, in turn, with negative outcomes such as depression and poorer post-breakup adjustment [23], [48]. Although these forms of rumination may hinder emotional recovery, we propose that the confrontation of loss through rumination may actually promote growth. Thus, given that breakup distress predicts intrusive thoughts about a former partner [37], and the experience of intrusive thoughts soon after a stressful event is related to reflection and greater posttraumatic growth [30], [49], [50], we surmised that ruminative brooding may actually be adaptive for promoting post-breakup growth. Similar to our hypothesis for reflection, we expected that brooding would be particularly associated with anxious individuals’ personal growth, but only when the breakup occurred longer ago. Finally, because attachment avoidance is associated with the defensive suppression of attachment threats [13] and with less breakup-specific preoccupation [23], we predicted that highly avoidant individuals’ lower breakup distress would be associated with less brooding and reflection and, in turn, with lower personal growth.

### Rebounding

After a breakup, individuals who are higher in anxiety are more likely to turn to new romantic partners for a safe haven [21], which allows for the down-regulation of physiological dysregulation, the transfer of attachment needs to a new partner, and the restoration of felt security [45]. Indeed, highly anxious people tend to report less longing for an ex-partner insofar as they are involved in a “rebound” relationship or feel optimistic about their chances of finding a new partner [24]. In Study 2, we predicted that attachment anxiety and breakup distress would be associated with greater rebounding through heightened dating activity and/or casual sex, and attachment avoidance with less. We expected that rebounding, in turn, would be associated with greater personal growth.

### Methods

#### Participants

Data was collected from 473 participants (393 women, 75 men, 5 unspecified). They were recruited by posting links to an online survey on several psychology survey-hosting websites (Social Psychology Network Online Social Psychology Studies, Psychological Research on the Net, and the intranet at the authors’ university). The link to Study 2 was posted after the link to Study 1 was removed from all websites; nonetheless, to further ensure that the samples in Studies 1 and 2 were independent, the IP addresses of participants in both studies were compared for duplicates. 8 duplicates were removed, resulting in a sample size of 465 participants (385 women, 75 men, 5 unspecified; *M*_age_ = 21.36, *SD* = 5.49). 52% of participants were currently involved in a relationship; of these participants, 71% were exclusively dating their current partner, 8% were cohabitating, 8% were married, 7% were engaged, and 7% were casually or non-exclusively dating their current partner. 87% of participants were American, 4% were British, 3% were European, 2% were Latin American, 2% were Canadian, and the remaining 2% represented a variety of nationalities. There were no significant gender differences for any of the demographic variables.

#### Procedure and materials

The same measures used in Study 1 to assess attachment style, information about the former relationship and its demise, breakup distress, current distress, and personal growth were answered in the same order in Study 2. The following additional measures were completed after the measure of breakup distress. Cronbach’s alpha coefficients are reported in Table 2.

**Table 2:** Study 2: Descriptive statistics, Pearson’s correlations and Cronbach’s alpha coefficients.

Variable	1	2	3	4	5	6	7	8	9	10	11	12	13
1. Weeks since breakup	1.00												
2. Partner initiated	.03	1.00											
3. Relationship length	.06	−.02	1.00										
4. Currently involved	**.27**	−.01	.03	1.00									
5. Current distress	−.15**	.12*	.03	**−.33**	1.00								
6. Anxiety	−.10*	.13**	.10*	**−.25**	**.29**	1.00							
7. Avoidance	−.01	−.05	.01	**−.21**	.11*	**.43**	1.00						
8. Breakup distress	.02	**.38**	.11*	−.01	**.36**	**.36**	.03	1.00					
9. Brooding	−.01	**.27**	.06	.01	**.33**	**.36**	.06	**.80**	1.00				
10. Reflection	−.04	.02	.13**	.05	−.01	.10*	−.07	**.38**	**.47**	1.00			
11. Proclivity to rebound	−.06	−.01	.11*	−.03	.03	**.17**	.07	.11*	.14**	.13**	1.00		
12. Num. of new partners	**.32**	−.01	.10*	**.30**	**−.29**	−.05	−.03	.05	.02	.08†	**.21**	1.00	
13. Personal growth	.02	.02	.13**	**.19**	**−.22**	−.02	**−.22**	**.29**	**.31**	**.37**	.14**	**.18**	1.00
Mean	85.02	–	91.95	–	13.90	48.36	41.09	51.28	18.45	28.94	4.40	1.28	67.56
*SD*	113.54	–	171.46	–	6.65	14.67	13.24	17.56	6.85	7.88	2.37	1.41	22.45
α	–	–	–	–	.88	.92	.92	.95	.90	.85	.56	–	.96

† *p*<.10.

* *p*<.05.

** *p*<.01. Bolded figures were significant at *p*<.0001. Num. of new partners = number of new dating partners since the breakup.

#### Ruminative brooding and reflection

Saffrey and Ehrenberg’s [23] General Rumination Scale, consisting of six items that measure general tendencies to engage in brooding and four items that measure reflection, was adapted to assess breakup-specific brooding and reflection. Participants were asked to think back to the period following the breakup, and indicate how often they engaged in brooding (e.g., “How often did you get irritated with how much you were thinking about the past relationship and/or breakup but found you couldn’t stop yourself from doing so?”) and reflection (e.g., “How often did you reflect on your experiences in your former relationship to learn from them?”). Furthermore, we included the five general reflection items of the Ruminative Responses Scale [51], also adapted to measure breakup-specific reflection (e.g., “How often did you analyse your personality to try to understand why you felt the way you did about your former relationship?”). Responses were assessed with a 5-point Likert scale anchored with *Never/Not at all* (1), *Moderately often* (3), and *Very often* (5). This retrospective approach to measuring rumination soon after a distressing event has been adopted elsewhere [51].

#### Proclivity to rebound

Two items inspired by Davis and colleagues’ [21] inventory asked participants to indicate how often they had replaced their ex-partner with new partners and engaged in casual sex since the breakup. Responses were measured with a 5-point Likert scale anchored with *Never/Not at all* (1), *Moderately often* (3), and *Very often* (5). In Table 2, these two items were summed to form an index of proclivity to rebound. Participants were also asked to indicate how many people they had dated since the breakup.

### Results

#### Descriptive statistics

Means, standard deviations, zero-order correlations, and Cronbach’s alpha coefficients are reported in Table 2. Two gender differences were significant: men reported greater current distress over the breakup [*M*_men = _15.41, *SD* = 5.98; *M*_women_ = 13.55, *SD* = 6.72; *t*(454) = 2.21, *p*<.05], and greater proclivity to rebound [*M*_men = _5.09, *SD* = 2.56; *M*_women_ = 4.25, *SD* = 2.32; *t*(456) = 2.83, *p*<.01].

First, we examined the zero-order correlations to assess whether they met Baron and Kenny’s [38] preconditions for mediation. Similar to the findings of Study 1, there was a significant correlation of avoidance but not anxiety with personal growth. All of the proposed mediators (breakup distress, ruminative brooding and reflection, rebounding, and number of new partners since the breakup) were significantly correlated with personal growth. Anxiety was significantly correlated with most of these mediators, whereas avoidance was not. Avoidance significantly predicted breakup distress (β = −.14, *p*<.01) and personal growth (β = −.30, *p*<.0001) when anxiety was included as a predictor in the regression model. These associations remained significant after several of the control variables (sex, age, self-esteem, initiator status, length of time since the breakup, current involvement, length of the past relationship, and current distress) were included as covariates. Anxiety significantly predicted breakup distress (β = .42, *p*<.0001) and personal growth (β = .12, *p*<.05) when avoidance was included as a predictor. After the control variables were included as covariates, anxiety remained a significant predictor of breakup distress and personal growth. Furthermore, we controlled for ratings of past relationship quality in both regression models, but because the significance of avoidance, anxiety, and breakup distress as predictors of personal growth did not change, and we believed such ratings were particularly subjective post-breakup, we decided not to include this covariate in the analyses.

These analyses therefore provided at least some preliminary support for our theoretical model. To examine whether the data from Study 2 replicated the final model in Study 1 (Figure 1), we first tested the fit of a simplified model that only included breakup distress as a mediator of the attachment style – personal growth relationship. The fully saturated structural model provided a good fit to the data [χ^2^(48) = 72.22, *p* = .01, CFI = .99, RMSEA = .04 (CI = .02,.05), SRMR = .02]. Similar to Study 1, the direct effect of avoidance but not anxiety on personal growth was significant. A modified model that constrained the latter path to zero provided a good fit to the data [χ^2^(49) = 72.34, *p* = .02, CFI = 1.00, RMSEA = .04 (CI = .02,.05), SRMR = .02], and did not significantly differ from the initial model [χ^2^D(1) = .12, *p*>.05].

To test the full model, three item parcels, created via the method used in Study 1, were assigned to each of the latent variables. Rebounding, however, was measured with three indicator items rather than parcels – the two scale items assessing proclivity to rebound, and the number of people dated since the breakup. AMOS 18 was used to test the measurement model and the structural model. Because AMOS 18 requires complete data, the following analyses were based on 370 participants (308 women, 62 men).

#### Measurement model

A confirmatory factor analysis revealed that the data provided a good fit to the measurement model [χ^2^(168) = 314.84, *p*<.0001, CFI = .98, RMSEA = .05 (CI = .04,.06), SRMR = .05]. The loadings of the indicators on the appropriate latent variables were all significant (*p*<.0001).

#### Structural model

The structural model included all direct and indirect effects of anxiety, avoidance, and breakup distress on personal growth, but did not specify paths between brooding, reflection, and rebounding. This model tested breakup distress as a mediator of the associations of attachment anxiety and avoidance with brooding, reflection, and rebounding; it also tested brooding, reflection, and rebounding as mediators of the association of breakup distress with personal growth. The initial test of this model yielded a good fit to the data [χ^2^(171) = 364.14, *p*<.0001, CFI = .97, RMSEA = .06 (CI = .05,.06), SRMR = .06]. Inspection of the modification indices suggested that adding an error covariance between the latent variables of brooding and reflection – indicating that they overlapped considerably – would improve model fit. A modified model that included this error covariance provided a significantly better fit to the data [χ^2^(171) = 321.10, *p*<.0001, CFI = .98, RMSEA = .05 (CI = .04,.06), SRMR = .06, χ^2^D(1) = 43.04, *p*<.05].

**Figure 2:**
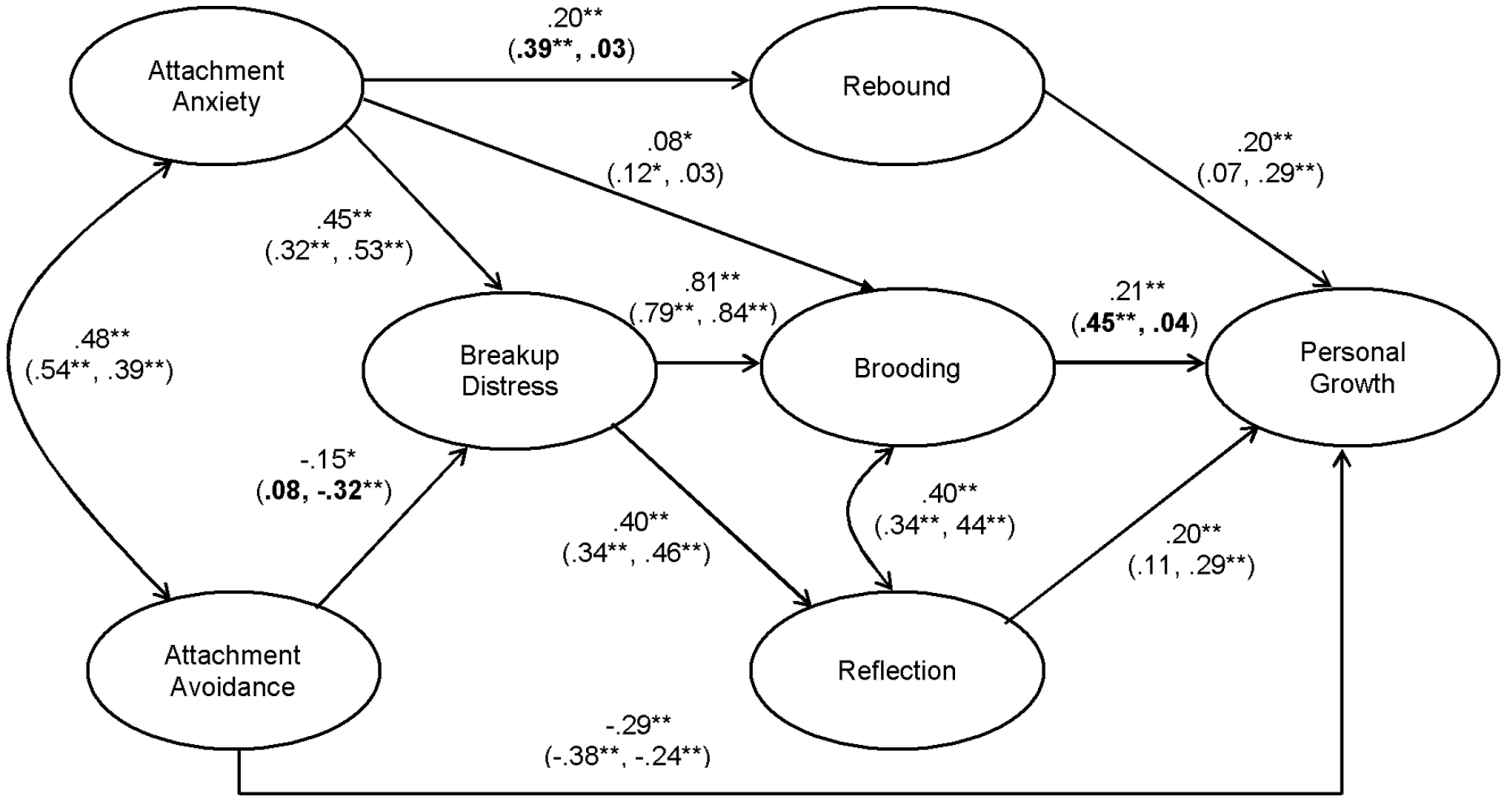
Study 2: Final model. The values within parentheses are the path coefficients for people whose breakup occurred longer ago (left side) and more recently (right side). Bolded values represent a significant group difference in the path coefficients.^ †^p<.10,*p<.05, **p<.01.

Several structural path coefficients were not significant, and were constrained to zero in descending order of the strength of their path coefficients: avoidance to rumination, breakup distress to rebounding, anxiety to reflection, avoidance to rebounding, breakup distress to personal growth, avoidance to reflection, and anxiety to personal growth. All remaining structural path coefficients were significant. The modified model with these non-significant paths constrained to zero yielded a good fit to the data [χ^2^(177) = 326.48, *p*<.0001, CFI = .98, RMSEA = .05 (CI = .04,.06), SRMR = .06], and did not significantly differ from the model without these paths constrained [χ^2^D(6) = 5.38, *p*>.05]. Because these paths did not significantly contribute to the model, the more parsimonious modified model was established as the final model. The path coefficients for this final model are reported in Figure 2.

There were two direct effects in the final model: from avoidance to personal growth, and from anxiety to brooding. When the path from avoidance to personal growth was constrained to zero, the fit of the model was significantly worse [χ^2^(178) = 358.02, *p*<.0001, CFI = .97, RMSEA = .05 (CI = .04,.06), SRMR = .08, χ^2^D(1) = 31.54, *p*<.05], indicating that this direct effect should be retained. Likewise, when the direct effect of anxiety on brooding was constrained to zero, the fit of the model was significantly worse [χ^2^(178) = 331.41, *p*<.0001, CFI = .98, RMSEA = .05 (CI = .04,.06), SRMR = .06, χ^2^D(1) = 4.93, *p*<.05], and so this direct effect was also retained.

### Tests of Indirect Effects

To assess the significance of the indirect effects in the final model, we examined the 95% bias-corrected confidence intervals of 1,000 bootstrap samples. The results indicated that anxiety was indirectly associated with personal growth through rebounding [β = .06, *p* = .003 (CI:.02,.13)] and brooding [β = .12, *p* = .002 (CI:.07,.19)]; anxiety was indirectly associated with brooding [β = .37, *p* = .001 (CI:.27,.46)] and reflection [β = .18, *p* = .001 (CI:.11,.26)] through breakup distress; avoidance, too, was indirectly associated with brooding [β = −.12, *p* = .02 (CI: −.23, −.02)] and reflection [β = −.06, *p* = .01 (CI: −.13, −.01)] through breakup distress; and breakup distress was indirectly associated with personal growth through brooding [β = .26, *p* = .002 (CI:.17,.35)] and reflection [β = .14, *p* = .002 (CI:.08,.22)].

### Multiple-Groups Comparison Analysis: Time since the Breakup

To compare model fit for participants whose breakup occurred longer ago (i.e., more than the median of 51.6 weeks; *N* = 182) with those whose breakup occurred more recently (less than the median; *N* = 189), we conducted a multiple-group comparison analysis with AMOS 18. Preliminary *t*-tests showed that the groups did not significantly differ in the variables rated retrospectively (breakup distress, reflection, and rumination), nor in personal growth. Similar to Study 1, participants whose breakup occurred more recently relative to those whose breakup occurred longer ago were significantly greater in anxiety [*M*s = 51.07 and 45.79, *SD*s = 14.74 and 14.15, respectively; *t*(421) = 3.75, *p*<.0001], avoidance [*M*s = 42.43 and 39.53, *SD*s = 13.45 and 12.88, respectively; *t*(426) = 2.28, *p*<.05] and current distress [*M*s = 16.25 and 11.27, *SD*s = 6.88 and 5.41, respectively; *t*(450) = 8.51, *p*<.0001]. Participants whose breakup occurred longer ago reported dating significantly more people since the breakup relative to those whose breakup was more recent [*M*s = 1.84 and 0.76, *SD*s = 1.46 and 1.15, respectively; *t*(448) = 8.73, *p*<.0001].

To test whether the final model (Figure 2) fit the data similarly in the two groups, multiple-group comparison analysis first revealed that the factor loadings did not significantly differ across groups [χ^2^D(14) = 15.25, *p*>.05], supporting the equivalence of the measurement model. Second, given invariant factor loadings, the model in which the structural path coefficients were constrained to invariance across groups significantly differed from the model in which these paths were free to vary [χ^2^D(10) = 35.77, *p*<.0001], indicating that at least one of the path coefficients was not equal across groups. To locate which paths were not equal, each structural path was individually constrained to invariance and compared against the model in which all structural paths were free to vary. Due to the inflated risk of Type I errors, alpha was set at.01. Only three paths were not invariant across groups: avoidance to breakup distress [χ^2^D(1) = 10.49, *p*<.01], which was significant for people whose breakups occurred more recently (*β* = −.32, *p*<.01), but not for people whose breakups occurred longer ago (*β* = .08, *ns*); anxiety to rebounding [χ^2^D(1) = 7.68, *p*<.01], which was significant for people whose breakups occurred longer ago (*β* = .39, *p*<.01), but not for people whose breakups occurred more recently (*β* = .03, *ns*); and ruminative brooding to personal growth [χ^2^D(1) = 11.00, *p*<.01], which was significant for people whose breakups occurred longer ago (*β* = .45, *p*<.01), but not for people whose breakups occurred more recently (*β* = .04, *ns*). Path coefficients for each group are reported in parentheses in Figure 2.

### Summary

The results of Study 2 replicated the results of Study 1: attachment anxiety was positively associated with personal growth, and attachment avoidance negatively associated, because of intensified and inhibited breakup distress, respectively. As hypothesized, brooding and reflection mediated the link between breakup distress and personal growth. Anxiety was also indirectly associated with personal growth through brooding and the proclivity to rebound. Finally, the multiple-group comparison suggested that a considerable period of time needed to elapse after the breakup before highly anxious people rebounded with new partners, and before ruminative brooding encouraged greater personal growth; conversely, avoidant individuals only appeared to suppress their breakup distress when the breakup was more recent.

## General Discussion

Taken together, these studies provide substantial evidence that attachment-anxious individuals experience greater personal growth following romantic breakups, and attachment-avoidant individuals less, through the mechanisms of breakup distress, rumination, and rebounding with new partners. Arguably, these findings suggest that the pain of breakups has the potential to exert a transformational effect on anxious but not avoidant individuals. We review key findings below.

First, both studies revealed that attachment anxiety was associated with greater breakup distress, and avoidance with less, consistent with other research [1], [21], [25], [26], [28]. These differences in distress reflect anxious and avoidant individuals’ tendencies toward attachment system hyperactivation versus deactivation, respectively [13]. Furthermore, breakup distress was positively associated with personal growth, in line with studies linking the intensity of peritraumatic distress with posttraumatic growth [34], [35]. Study 2 clarified two mediators – ruminative reflection and brooding – of this association. These forms of rumination may encourage the construction of meaningful narratives that help to promote personal growth [30], [47], [49], [50]. That attachment anxiety was associated with greater reflection and brooding via breakup distress suggests that these individuals, who have negative self-views [10] and a concomitant tendency to blame themselves for relationship dissolution [21], may scrutinize their self-perceived shortcomings in the aftermath of an upsetting breakup. This self-reflection may then motivate a course of self-improvement, perhaps in a bid to pre-empt the dissolution of future relationships. Meanwhile, attachment avoidance was related to lower reflection and brooding via breakup distress, suggesting that the defensive maintenance of positive self-views and the inhibition of breakup-specific thoughts and feelings deprives these individuals of an opportunity to look honestly at themselves and take stock of ways that they may improve themselves for the better.

Contrary to our hypothesis, breakup distress was not associated with proclivity to rebound; rather, rebounding directly mediated anxious individuals’ personal growth. This finding extends the work of Spielmann and colleagues [24], who found that highly anxious individuals were less likely to remain attached to an ex-partner insofar as they perceived new romantic prospects. To the extent that rebound relationships encourage attachment reorganization and detachment, anxious individuals’ cognitive and emotional resources may be diverted from the former partner into self-cultivation, potentially increasing their own attractiveness as a dating partner. Fear of further relationship failure may also motivate anxious individuals to develop their relationship maintenance skills within new relationships by carefully attending to their past relationship mistakes. Alternatively, highly anxious people may re-frame the past relationship as particularly unsatisfying when they enter a new relationship, thereby enhancing their sense of growth and being in a better place.

Overall, these results shed light on anxious and avoidant individuals’ potential for recovery and growth following relationship breakups. Insofar as attachment reorganization requires an oscillation between attachment system hyperactivation (to find meaning in loss) and deactivation (to down-regulate physiological dysregulation and enable emotional recovery) [13], [18], the present findings suggest that anxious individuals’ sustained attachment hyperactivation after a breakup may enable meaning-making and personal growth, even if it comes at the cost of full emotional recovery. Indeed, highly anxious participants in Studies 1 and 2 reported greater current distress over the breakup in spite of their greater personal growth. That posttraumatic growth is weakly or not associated with well-being [30], [50] is a further reminder that personal growth is not a panacea. Avoidant individuals’ deactivation, on the other hand, may facilitate emotional recovery, but at the expense of cultivating a meaningful narrative and positive changes in one’s life.

The cross-sectional approach adopted in these studies allowed us to roughly discern the time course by which people may transform breakup distress into personal growth. In Study 2, the path between brooding and personal growth was significant for people whose breakup occurred longer ago, but not for people whose breakup occurred more recently. Similarly, in Study 1, the link between breakup distress and personal growth was only significant for people whose breakup occurred longer ago (this difference in coefficients was not significant, however, and should only be interpreted cautiously). Moreover, anxious individuals in Study 2 were only more likely to go on the rebound after sufficient time had passed since the breakup, suggesting that the initial blow of more recent breakups may temporarily neutralize their tendency to seek new partners. Consistent with the proverb that “time heals all wounds,” these findings suggest that a substantial amount of time may be needed to sublimate breakup distress, ruminative brooding, and rebounding with new partners into personal growth.

### Limitations and Future Directions

A notable limitation of these studies is that the ratings of breakup distress and rumination were retrospective, and therefore susceptible to memory bias. Accordingly, the multiple-group comparisons found that when the breakup was more distal versus more proximal, anxious participants reported less breakup distress, whereas avoidant participants reported more. Thus, time may blunt anxious individuals’ memories of their breakup distress, or erode avoidant individuals’ suppression of their breakup-related memories. We do not consider these findings to be incompatible with our theorizing, however; in fact, these findings merely underscore the tendencies of anxious and avoidant individuals to hyperactivate or deactivate the attachment system, respectively, in the face of proximal threats. It is also important to note that participants whose breakup occurred longer ago versus more recently did not significantly differ on the variables rated retrospectively (breakup distress, brooding, and reflection), nor were there many group differences in the pattern of associations, suggesting that memory bias did not strongly influence our findings. Moreover, asking participants to write about the circumstances of their breakup may have reduced memory bias on subsequent measures by priming memories of feelings experienced at the time of the breakup.

Nevertheless, a prospective longitudinal design that tracked reactions after a breakup, such as the daily diary design utilized by Sbarra and colleagues [1], [20] or one that collected follow-up data over several years, would further test our theoretical model and establish how much time is required to work through negative emotions via a process of ruminative brooding and reflection before personal growth may occur. Measuring psychological characteristics before and after a breakup could also compare set point theories of relationship loss, which equate recovery with the return to baseline levels of well-being [45], with stress-related growth perspectives, which propose that people may actually exceed their baseline through experiencing positive changes in their lives [29]. Until then, we cannot dismiss the possibility that perceived personal growth may not be accompanied by actual or lasting change, or even worse, that the strain of a painful breakup may actually intensify attachment insecurity, decrease well-being, and weaken resilience to relationship loss in the long run. Indeed, one longitudinal study found that people showed a permanent reduction in life satisfaction after a divorce [52]. Future research should therefore establish whether the post-breakup functioning of attachment-anxious individuals actually exceeds their baseline over time, as the present findings suggest, or whether it adapts to pre-breakup levels or even decreases.

Two other limitations of these studies are worth noting. First, we did not directly address the influence of attachment security on breakup distress and personal growth. Although attachment security can be inferred by low scores in anxiety and avoidance, this approach tends to sacrifice measurement precision [16]. If future studies include a more direct measure of attachment security, such as the Relationship Scales Questionnaire [53], we would predict that secure individuals would report less breakup distress than anxious individuals, but more than avoidant individuals; consequently, we would expect secure individuals to report less personal growth than anxious individuals, but more than avoidant individuals.

Second, the participant samples in these studies may have been self-selected, potentially reducing the generalizability of our results. Because participants were not compensated for completing the surveys, it is possible that individuals were particularly motivated to take part because they had experienced difficult breakups. Still, while self-selection may have driven up the scores on some of the measures, we have no reason to believe that the pattern of associations that emerged in these studies would differ for individuals whose breakups were slightly less distressing.

### Concluding Remarks

Friedrich Nietzsche once wrote, “That which does not kill us makes us stronger.” Accordingly, the present research suggests that the pain of breakups might eventually lead some people to grow and develop into stronger, wiser, and more self-cultivated individuals. Theoretically, these findings establish that individual differences in attachment style contribute to personal growth following relationship loss. More practically, the current results suggest that people who have recently experienced a breakup might benefit from working through their emotional distress, particularly via ruminative reflection and considering relationships with new partners. Ruminative brooding might also promote personal growth, but only after the acute emotional distress experienced immediately after a breakup has subsided. Overall, this research suggests that a broken heart has the potential to motivate positive self-transformation, especially in the individuals who have suffered the most.
